# Effective Thallium(I) Removal by Nanocellulose Bioadsorbent Prepared by Nitro-Oxidation of Sorghum Stalks

**DOI:** 10.3390/nano12234156

**Published:** 2022-11-24

**Authors:** Hui Chen, Priyanka R. Sharma, Sunil K. Sharma, Abdulrahman G. Alhamzani, Benjamin S. Hsiao

**Affiliations:** 1 Department of Chemistry, Stony Brook University, Stony Brook, NY 11794-3400, USA; 2 Department of Chemistry, Imam Mohammad Ibn Saud Islamic University (IMSIU), Riyadh 11632, Saudi Arabia

**Keywords:** nitro-oxidation, thallium removal, cellulose nanofibers, sorghum stalks

## Abstract

Thallium(I) (Tl(I)) pollution has become a pressing environmental issue due to its harmful effect on human health and aquatic life. Effective technology to remove Tl(I) ions from drinking water can offer immediate societal benefits especially in the developing countries. In this study, a bio-adsorbent system based on nitro-oxidized nanocellulose (NOCNF) extracted from sorghum stalks was shown to be a highly effective Tl(I) removal medium. The nitro-oxidation process (NOP) is an energy-efficient, zero-waste approach that can extract nanocellulose from any lignocellulosic feedstock, where the effluent can be neutralized directly into a fertilizer without the need for post-treatment. The demonstrated NOCNF adsorbent exhibited high Tl(I) removal efficiency (>90% at concentration < 500 ppm) and high maximum removal capacity (*Q_m_* = 1898 mg/g using the Langmuir model). The Tl(I) adsorption mechanism by NOCNF was investigated by thorough characterization of NOCNF-Tl floc samples using spectroscopic (FTIR), diffraction (WAXD), microscopic (SEM, TEM, and AFM) and zeta-potential techniques. The results indicate that adsorption occurs mainly due to electrostatic attraction between cationic Tl(I) ions and anionic carboxylate groups on NOCNF, where the adsorbed Tl(I) sites become nuclei for the growth of thallium oxide nanocrystals at high Tl(I) concentrations. The mineralization process enhances the Tl(I) removal efficiency, and the mechanism is consistent with the isotherm data analysis using the Freundlich model.

## 1. Introduction

Thallium (TI) pollution has become a major environmental challenge. Around 60% of Tl contaminants come from the electronic industry, and the rest comes from the pharmaceutical and glass manufacturing industries [1]. Alleviated levels of Tl exposure over time in terrestrial, aerial, and aquatic systems can greatly harm human health, [2] where the major human exposure to Tl is through drinking water. When Tl compounds enter the bloodstream, they can be transported through many organs and accumulate in bones, kidneys, and the nervous system [3]. As a result, the functioning of some essential enzymes can be disrupted. The classic syndromes of Tl poisoning include stomach and intestinal ulcers, alopecia, and polyneuropathy. Other symptoms include astral disorders, insomnia, paralysis, loss of body mass, internal bleeding, myocardial injury, and, in the most severe case, death [4,5,6,7,8].

Tl exists in two main oxidation states in the aquatic environment: Tl(I) and Tl(III), where Tl(I) is the stable and mobile form, and Tl(III) is the unstable and reactive form. Tl(III) can be easily reduced to Tl(I) with reducing agents or via hydrolyzation in alkaline and neutral conditions [9]. This indicates that Tl(I) is the dominant species in thallium pollution in natural water and industrial wastewater [10]. Furthermore, the removal of Tl(I) has been found to be more difficult than the removal of Tl(III) [11]. Tl(I) exhibits high toxicity; therefore, the upper limit of Tl(I) concentration in drinking water has been set at 2 μg/L in the U.S. [4,12,13]. In recent years, many approaches have been demonstrated for the removal of Tl(I) from water, such as enhanced coagulation and adsorption, ion exchange, electrodialysis, and reverse osmosis (RO) methods [4,14,15,16]. Some methods exhibit some notable shortcomings in practice. For example, the ion exchange method can be easily affected in real environmental conditions, and the column format cannot be used for long-term operation [13]. The RO approach, although effective, is energy-intensive, and the spent RO membranes cannot be easily recycled and reused [13,17]. Among these methods, the coagulation/adsorption approach is widely recognized as an efficient and cost-effective method due to its simplicity and high removal capacity [18,19]. Additionally, while various absorbents have been demonstrated for Tl removal from wastewater, e.g., MnO_2_ [20], TiO_2_ [21], polyacrylamide [22], multi-walled carbon nanotubes [23], powdered eucalyptus leaves [24], and sawdust [25], the cost-effectiveness, removal efficiency and sustainability of these adsorbents are still not ideal.

To overcome the concern of cost-effectiveness, we are particularly interested in the use of underutilized biomass feedstocks, such as agriculture residues, paper, and cardboard waste as resources to extract valuable nanomaterial for water purification due to their up-cycling potential and abundance. In this regard, we aim to demonstrate that nanocellulose can be an effective coagulant/adsorbent to remove the Tl(I) contaminant from water [26]. In our previous study, we demonstrated that carboxylated cellulose nanofibers (CNFs) are generally effective for heavy metal ions removal [27,28,29,30,31,32]. Furthermore, anionic CNF can also be converted into cationic CNF through surface modification to remove metal ions in the anionic form. For instance, thiol-group functionalized CNF was reported to be an effective adsorbent for arsenic ions (As(III)) [33] and chromium ions (Cr(VI)) [34,35]. As carboxylated CNF are effective to remove most heavy metal ions in the cationic form directly, such as lead ions (Pb(II)) [36], uranium ions (U(VI)) and uranyl ions (UO_2_^2+^), [37,38] we aim to demonstrate the use of another form of low-cost carboxylated CNF as an effective adsorption medium to remove Tl(I).

Various chemical methods have been used to extract CNFs from biomass feedstock, including TEMPO mediated oxidation [39,40,41,42,43], carboxymethylation [44], phosphorylation [45], acetylation [46] and silylation [47]. However, most of these methods contain multiple steps including the pulping pretreatment (i.e., remove lignin and hemicellulose, and subsequent cellulose oxidation process.) In our lab, we combined the treatments of pulping and cellulose oxidation [48,49] in a simple approach, termed nitro-oxidation process (NOP), using nitric acid (HNO_3_) or nitric acid-sodium nitrite (NaNO_2_) mixtures to extract CNF from any untreated biomass. The CNF obtained NOP is termed as NOCNF. The mechanism of NOP for CNF extraction is that the presence of HNO_3_ can degrade (thus remove) lignin and hemicellulose in the biomass, while nitrosonium (NO^+^) can be generated to oxidize the primary hydroxyl group (−CH_2_OH) at the C6 position to create carboxyl groups on cellulose. As the effluent containing degraded lignin and hemicellulose components, unreacted nitric acid, and nitrate products, can be efficaciously neutralized into slow-release fertilizers; the NOP approach is a zero-waste pathway, where no recycling or reuse of chemicals is needed [48]. Compared with the conventional methods to extract nanocellulose from raw biomass, the NOP approach greatly reduces the need for multichemical, water and electric energy, and is particularly suited to extract nanocellulose from non-woody biomass with low lignin content and loose cell wall structure. For this reason, this study chose sorghum stalk as the biomass feedstock. This is because sorghum is a major food crop in many parts of the world (e.g., many countries in Africa, India, and China), however, its residue after harvest is vastly underutilized. As thallium contamination has also become a growing issue in these countries [50], the use of NOCNF from sorghum stalks to remove Tl(I) ions from drinking water can offer immediate societal benefits. 

In this study, we developed a highly efficient adsorbent for Tl(I) removal by extraction of carboxylated cellulose nanofiber directly from untreated sorghum stalks using the nitro-oxidation method. The Tl(I) concentration after the NOCNF adsorption treatment was measured by inductively coupled plasma mass spectroscopy (ICP-MS). Additionally, the mechanism of Tl(I) removal with NOCNF was investigated by Fourier-transform infrared spectroscopy (FTIR), scanning electron microscopy (SEM)/energy dispersive X-ray spectroscopy (EDS), transmission electron microscopy (TEM)/electron diffraction (ED), atomic force microscopy (AFM), and wide-angle X-ray diffraction (WAXD) techniques. To explore the feasibility of using NOCNF (derived from sorghum stalks) in large scale removal of Tl(I) from water, the separation column containing freeze-dried NOCNF solid samples was used. It has been reported that the microporous structure in freeze-dried bioadsorbent can exhibit fast mass-transfer kinetics in a way that decreases mass-transfer resistance [51]. Moreover, the sponge-like structure in the freeze-dried sample can provide plentiful adsorption sites due to the high surface area, thus enabling high water purification performance [52]. This expectation was tested by using the gravity-driven column adsorption method.

## 2. Materials and Methods

### 2.1. Materials

Sorghum stalk samples were obtained from a farm in Botswana, Africa. Nitric acid (ACS reagent, 70%), sodium nitrite (ACS reagent ≥ 97%), thallium acetate (Tl(Ac)), sodium hydroxide, and hydrochloric acid (36% assay) were purchased from Fisher Scientific (Waltham, MA, USA). All the chemicals were used without further purification.

### 2.2. NOP Extraction of Cellulose Nanofibers (NOCNF) from Sorghum Stalks

Carboxylated cellulose nanofibers were extracted from sorghum stalks by the nitro-oxidation process (NOP) [48]. In brief, 2 g of raw sorghum stalks in the granular form was placed in a three-neck round bottom flask, where 28 mL of nitric acid (65 wt%, 0.24 mol) was added slowly into the flask until the fibers were completely soaked and pulped. Subsequently, 0.028 mol (1.932 g) of sodium nitrite was added to the reaction mixture under continuous stirring (red fumes (NO_2_) were formed immediately upon the sodium nitrite addition). To prevent the escape of red fumes, the mouths of the flask were completely sealed by parafilm. The reaction was carried out at 50 °C for 12 h under stirring and was subsequently quenched by adding 250 mL of distilled water. The product was rested properly where the upper portion was decanted off to remove the excess of acid. The decantation step was repeated 2–3 times, until the solid residues started to suspend in water. Next, the slurry was centrifuged at 3000 rpm for 10 min, until the pH of the upper suspension reached above 2.5. The suspension was then transferred to a dialysis bag (6–8 kDa) for further purification. The dialysis was continued for 4–5 days, until the conductivity of water reached below 5 µS. After dialysis, the slurry was again centrifuged at 3000 rpm for 5 min, to separate microfibers and nanofibers. The carboxyl group (-COOH) on the cellulose nanofiber (CNF) was converted to the carboxylate group (-COONa) by treatment with 8% sodium bicarbonate, followed by dialysis again. Finally, the pH value of the NOCNF suspension was about 5.3 due to the presence of unconverted carboxyl groups in CNF.

### 2.3. Characterization of NOCNF and NOCNF-Tl Floc

The conductometric titration method was used to determine the carboxylate content in NOCNF [40]. The detailed description of the titration procedure is discussed in Supporting Information. Moreover, the surface charge of the NOCNF was determined by measuring the zeta-potential using Zetaprobe Analyzer^TM^ (Colloid Dynamics LLC, Ponte Vedra Beach, FL, USA). The structural and functional characterizations of NOCNF and NOCNF-Tl floc were performed using Fourier-transform infrared (FTIR) in the attenuated total reflectance (ATR) mode (PerkinElmer, Waltham, MA, USA), wide-angle X-ray diffraction (WAXD, Benchtop Rigaku MiniFlex 600), and thermogravimetric analysis (TGA) using STA-6000 Simultaneous Thermal Analyzer (PerkinElmer, Waltham, MA, USA). The morphology of NOCNF was analyzed by transmission electron microscopy (TEM) using FEI Tecnai G2 Spirit (BioTWIN, Quebec city, QC, CA), atomic force microscopy (AFM) using scanning probe microscope (Bruker, Billerica, MA, USA), and solution small-angle X-ray scattering (SAXS) using LiX beamline in NSLS-II in Brookhaven National Laboratory (Brookhaven, NY, USA). The surface area of freeze-dried NOCNF sample was measured by a Brunauer-Emmett-Teller (BET) analyzer (Anton Paar, Graz, Australia). Lignin and hemicellulose content were determined by the Celignis Biomass Analysis Laboratory (Limerick, Ireland). All above characterization methods and experimental conditions are elaborated in Supporting Information.

### 2.4. Static Thallium(I) Adsorption Study

A series of simulated Tl(I) solutions were made in the range of 10 to 500 ppm by dissolving the appropriate amount of thallium acetate (Tl(Ac)) into DI water (the final pH was 5.9). In the adsorption study, 2 mL Tl(I) solution was taken out and mixed with 2 mL NOCNF suspension. After 24 h contact time, the supernatant of the mixture was filtered by a 0.22 μm filter to remove the NOCNF-Tl floc. The filtrate was diluted to a proper concentration for ICP-MS measurement.

The static adsorption study of Tl(I) by NOCNF was performed by using the data from the ICP-MS analysis. The relationship between the adsorption capacity adsorbed at equilibrium (*Q_e_*) and the equilibrium concentration of the adsorbate (*C_e_*) was evaluated by both Langmuir isotherm model and Freundlich isotherm model. The Langmuir model assumes monolayer adsorption on the active adsorbing site, whereas the Freundlich model assumes multilayer adsorption. The Langmuir isotherm model can be expressed by Equation (1):

(1) $$\frac{C_{e}}{Q_{e}}=\frac{C_{e}}{Q_{m}}+\frac{1}{KQ_{m}}$$

where *Q_m_* is the maximum adsorption capacity, and *K* is the Langmuir constant, both can be calculated from the intercept and the slope of the linear *C_e_/Q_e_* versus *C_e_* plot, respectively [53]. The Freundlich isotherm model can be expressed by Equation (2):

(2) $$logQ_{e}=\frac{1}{n}logC_{e}+logK_{f}$$

where *K_f_* and *n* are the characteristic constants of the system [54].

### 2.5. The pH Effect on Thallium(I) Adsorption

The pH effect on the Tl(I) adsorption performance by NOCNF was also evaluated. In this study, three different pH conditions (pH = 3, 5.9, 10) were created by adding 0.1 N HCl or 0.1 N NaOH solution into 500 ppm Tl(I) solution. Afterward, 2 mL resulting Tl(I) solution was mixed with 2 mL NOCNF suspension (0.32 wt%). After 24 h contact time, the mixture was filtered by a 0.22 μm filter, and the filtrate was diluted for ICP-MS test.

### 2.6. Adsorption Column Application Study

**Figure 1:**
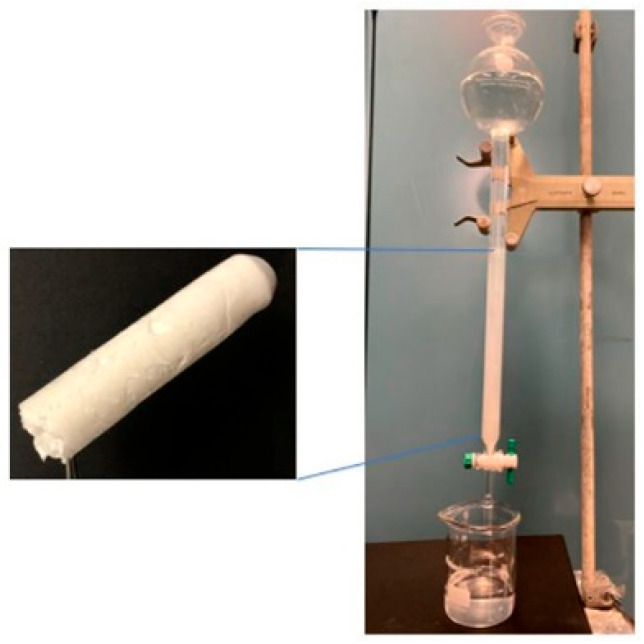
Freeze-dried NOCNF adsorbent and the adsorption column setup.

To prepare the adsorption column, NOCNF suspension was freeze-dried and stacked into a glass column (3 cm in diameter and 25 cm in length). Next, thallium(I)-contaminated water (100 ppm) was placed in the upper part of the column and passed through the column gradually under gravity (Figure 1). The filtrate water from the column was collected at varying times, and its Tl(I) concentration was determined by the ICP-MS analysis. The flow rate for the adsorption column study was kept at 20 mL/min.

## 3. Results and Discussion

### 3.1. Characterization of NOCNF

The quantitative determination of the carboxylate group (-COO-) in NOCNF was carried out using the conductometric titration method, which content was around 0.69 mmol/g. This value is lower than other NOCNFs extracted from jute [48], probably due to the higher content of lignin and hemicellulose in sorghum stalks. The residue contents of lignin and hemicellulose in NOCNF measured 11.56% and 35%, respectively. The average surface charge of extracted NOCNF measured by the zeta potential method was −110 ± 5 mV, where NOCNF suspension exhibited the typical polyelectrolyte behavior (e.g., as indicated by the fluctuation in zeta potential and corresponding conductivity curves in Figure S1, Supporting Information). The relevant structure and surface properties of NOCNF extracted from sorghum stalks by using the nitro-oxidation process are summarized in Table 1. Additionally, the surface area of freeze-dried NOCNF was determined to be 9.79 m^2^/g by the BET method. This value was higher than that from the NOCNF sample derived from jute [37]. The BET adsorption graph is shown in Figure S2, Supporting Information.

### 3.2. The Tl(I) Adsorption Mechanism by NOCNF

**Figure 2:**
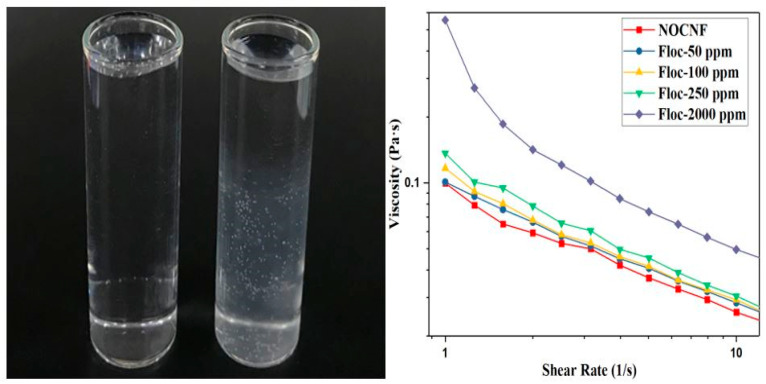
Photograph showing the NOCNF suspension (0.2 wt%) and the floc form by mixing NOCNF (0.2 wt%) with 100 ppm Tl(I). The viscosity of the floc sample increases with the increasing Tl(I) concentration.

Upon the mixing of NOCNF suspension and Tl(I) solution, a gel-like floc was formed immediately. An example floc formation is illustrated in Figure 2, where a 0.2 wt% NOCNF suspension was mixed with a 100 ppm Tl(I) solution. The floc could be seen in the cloudy form, which eventually precipitated to the bottom of the vial. Floc formation is initiated due to the electrostatic interaction between the COO- group on NOCNF and Tl(I) ions. 

Floc formation could significantly increase the viscosity of the suspension. In a rheological study, four floc samples were prepared by mixing NOCNF (0.2 wt%) with Tl(I) solution at concentrations of 50 ppm, 100 ppm, 250 ppm and 2000 ppm, respectively. The results are shown in Figure 2. It was seen that viscosity of the floc sample increases with the increasing Tl(I) concentration. This could be due to two reasons: (1) the pseudo-cross-linking effect between NOCNF and Tl ions (we note NOCNFs can be cross-linked by multivalent ions as Tl(III), while they can only be screened by monovalent ions as Tl(I)); and (2) the increased crowding effect due to the rising NOCNF aggregation tendency as the NOCNF surface becomes screened. It was interesting to note that all viscosity profiles of pure NOCNF suspension and NOCNF-TL flocs (gel) showed a shear thinning behavior.

**Figure 3:**
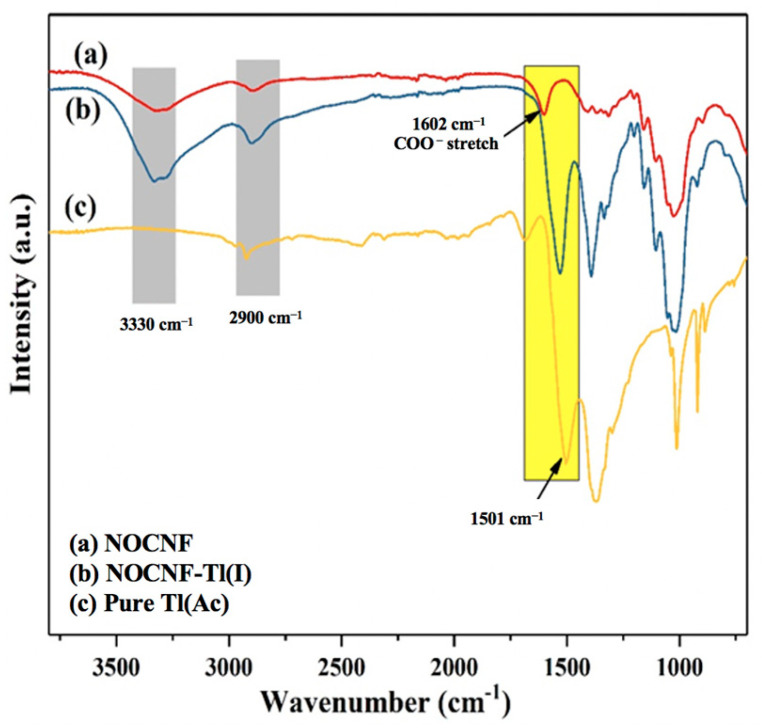
FTIR spectra of (**a**) NOCNF (0.32 wt%), (**b**) NOCNF-Tl (0.32 wt% NOCNF with 300 ppm Tl(I)), and (**c**) pure Tl(Ac).

To deeply investigate the interaction between NOCNF and Tl(I), the floc was obtained by mixing NOCNF (0.32 wt%) with 300 ppm Tl(I) solution. The FTIR spectra of NOCNF, NOCNF-Tl floc and pure Tl(Ac) are shown in Figure 3. The NOCNF spectra exhibited several characteristic peaks of cellulose: 3330 cm^−1^ for hydroxyl (−OH) stretching, 2900 cm^−1^ for CH symmetrical stretching [37]. The peak shown at 1602 cm^−1^ was assigned to COO- stretch, which is the characteristic peak for carboxylated CNF after the oxidation reaction (e.g., NOP). In the spectra of floc sample (Figure 3b), the peaks at 3330 cm^−1^ and 2900 cm^−1^ were found to become stronger, which may be due to a higher water content in the floc. It was also observed that the peak corresponding to carboxylate groups was shifted from 1602 cm^−1^ to 1540 cm^−1^, which could be explained by the electrostatic interaction between the COO- groups and Tl(I) ions, when Tl(I) adsorption happened.

**Figure 4:**
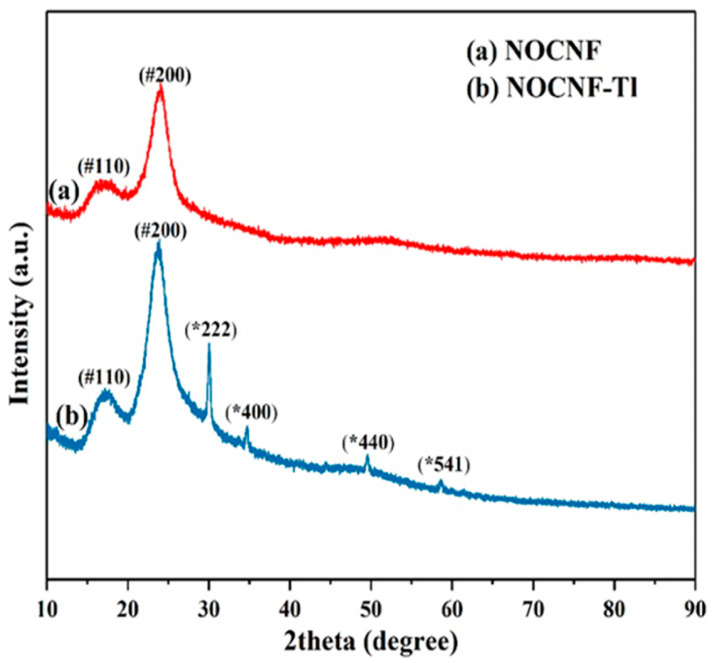
WAXD spectra of (**a**) NOCNF (0.32 wt%), and (**b**) NOCNF-Tl floc (mixed by 0.32 wt% NOCNF and 300 ppm Tl(I)); # represents the diffraction peaks from the cellulose I structure, * represents the diffraction peaks from the Tl_2_O_3_ crystal structure.

Wide-angle X-ray diffraction (WAXD) was employed to investigate the change in crystallinity of NOCNF (0.32 wt%) and NOCNF-Tl floc samples. The WAXD profile of NOCNF (Figure 4a) showed a diffraction pattern with peak positions at 2θ angles of 16.7, 24.2°, corresponding to the (110), (020) lattice planes of cellulose I structure, respectively [48]. Its crystallinity index (CI) was found to be about 43.5%, which was estimated from WAXD profile by Equation (S1) (Supporting Information). In contrast, the WAXD profile of NOCNF-TL floc exhibited several more distinct diffraction peaks at 30, 35, 50 and 58°, which can be assigned to the (222), (400), (440) and (541) lattice planes of thallium(III) oxide (Tl_2_O_3_) crystal structure [55,56]. The presence of Tl_2_O_3_ in the floc sample indicated that Tl(I) ions were successfully absorbed on the NOCNF surface, which were subsequently converted to Tl(III) when reacted with molecular oxygen and formed insoluble Tl_2_O_3_ crystal. The mineralization process follows the typical pathway of nucleation and growth with adsorbed Tl(I) behaving as a nucleus. It was found that the CI value of NOCNF after Tl adsorption remained the same (about 44%), which indicated the structure of NOCNF scaffold was not affected by the adsorption process.

**Figure 5:**
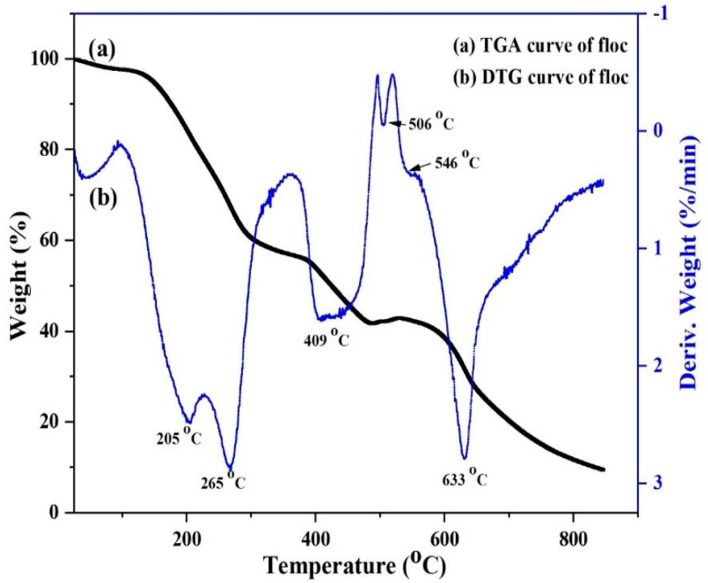
TGA and DTG spectra of the NOCNF-Tl floc (0.32 wt% NOCNF with 300 ppm Tl(I)).

The thermostability of the floc samples was also investigated, where the TGA and corresponding differential thermogravimetry (DTG) results are shown in Figure 5. The thermal degradation curve obtained for NOCNF-Tl floc exhibited a T_onset_ degradation temperature at 116 °C, which was lower than that of NOCNF (164 °C in our previous study [46]). It is well known that the initialization of thermal degradation in CNF is originated from the thermal degradation of the anhydroglucuronic acid unit. The lower T_onset_ degradation temperature in NOCNF-Tl floc was probably due to the instability of the carboxylate group in the COO-Tl linkage. Moreover, the T_offset_ value of the floc sample was found to be around 528 °C having 9 wt% final residues. This can be explained by the existence of residual thallium compounds. The DTG curve of the floc sample in Figure 5b contains four main peaks, in which the lowest peak was at 205 °C, corresponding to the degradation of the anhydroglucoronic unit.

The carboxyl group in the anhydroglucoronic unit is sensitive to thermal treatment due to facile decarbonization upon heating. Another peak adjacent to the degradation of the anhydroglucoronic unit was seen at 265 °C, indicating that the floc sample possessed a similar semi-crystalline structure as pure NOCNF. The peak at 409 °C can be assigned to the trace amount of the lignin residue from NOCNF. Since the thermal decomposition of Tl_2_O_3_ crystal takes place in the temperature range of 500–1000 °C, we speculate that the DTG peaks at 506, 546 and 633 °C may correspond to the degradation of Tl_2_O_3_ nanocrystals.

**Figure 6:**
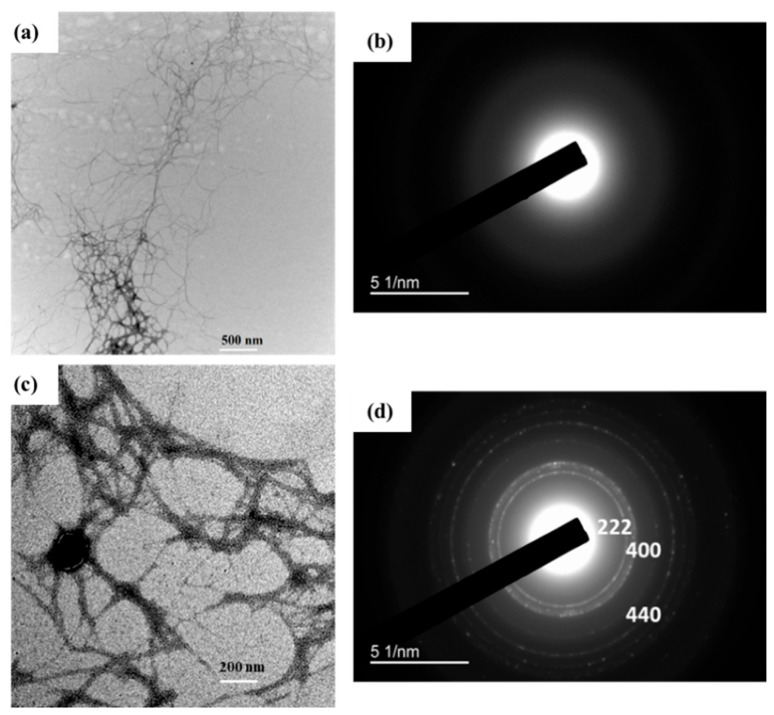
TEM and electron diffraction (ED) of (**a**,**b**) NOCNF (0.32 wt%)); (**c**,**d**) TEM and ED of NOCNF-Tl floc (0.32 wt% NOCNF with 300 ppm Tl(I)).

**Figure 7:**
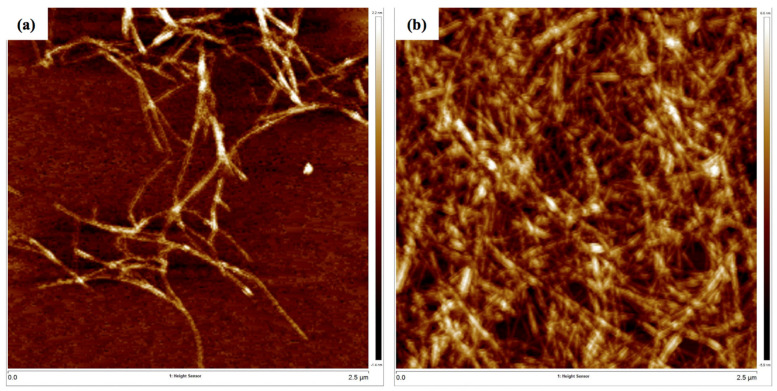
AFM images of (**a**) NOCNF (0.32 wt%) and (**b**) NOCNF-Tl floc (0.32 wt% NOCNF with 300 ppm Tl(I)).

Figure 6 shows a representative TEM image of extracted NOCNF from untreated sorghum stalks and NOCNF-Tl floc samples. In Figure 6a, the image revealed the fiber dimensions of NOCNF. Using the Image J software (National Institutes of Health, Bethesda, MD, USA) the average length of individual fiber was 1390 ± 380 nm and the average width was 8.53 ± 2 nm, which are comparatively higher than the NOCNF extracted from other biomass feedstocks, such as jute [46]. After the Tl(I) adsorption, the fibers became more entangled/aggregated (Figure 6b), showing the tightening of the network structure due to the interaction between NOCNF and thallium(I) ions. It was seen that black dots were formed amid the aggregation area, where these dots were identified to be thallium oxide nanocrystals, as confirmed by the ED pattern (Figure 6d) showing characteristic diffraction peaks of Tl_2_O_3_ (strong rings belonging to (222), (400), (440) lattice planes of the Tl_2_O_3_ crystal structure) [57]. These results are consistent with the WAXD results in Figure 4. The morphology of NOCNF and NOCNF-Tl floc was also characterized by the AFM technique, where two representative images are shown in Figure 7. In this figure, the thickness of NOCNF in the pure fibrous network and the floc could be determined by the vertical displacement using the AFM tapping mode measurement. The thickness of NOCNF was found to be 1.56 ± 0.8 nm, and it became 4.13 ± 0.5 nm after the Tl(I) adsorption. This confirmed the aggregation of NOCNF due to the electrostatic interaction between Tl(I) and NOCF.

**Figure 8:**
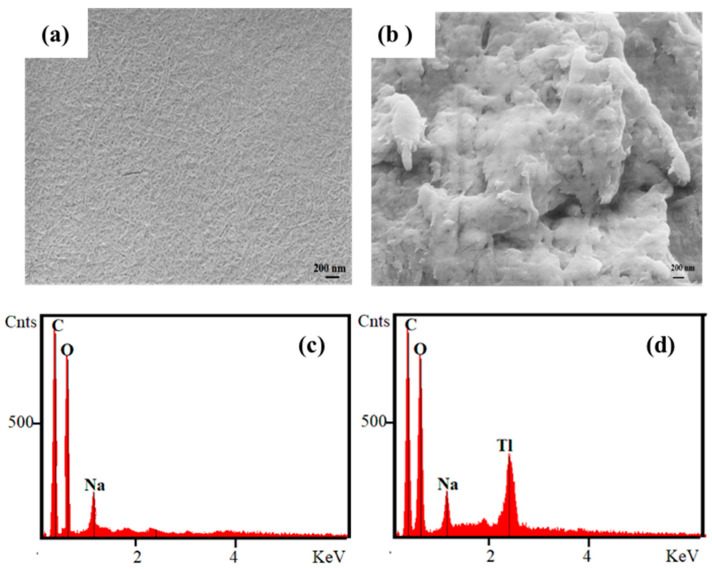
SEM of (**a**) NOCNF (0.32 wt%) and (**b**) NOCNF-Tl floc (0.32 wt% NOCNF with 300 ppm Tl(I)). Corresponding EDS spectra of (**c**) NOCNF (0.32 wt%) and (**d**) NOCNF-Tl floc (0.32 wt% NOCNF with 300 ppm Tl(I)).

SEM images of NOCNF and NOCNF-Tl floc (Figure 8) were also measured to provide some information regarding the morphological change before and after Tl(I) adsorption. In Figure 8, the SEM image of the floc obtained by mixing 300 ppm Tl(I) solution and NOCNF suspension (0.32 wt%) revealed a rough and patchy film with cloudy aggregates on the surface of the film, in comparison to a relatively uniform and smooth surface of the NOCNF film. The corresponding EDS spectra in Figure 8 provided the composition information of different elements in NOCNF and the floc. In neat NOCNF, only the carbon (C), oxygen (O), and sodium (Na) peaks were seen, which was consistent with the presence of the carboxylate group (−COONa) in NOCNF. In contrast, an intense Tl peak was detected in the NOCNF-Tl floc.

### 3.3. Tl(I) Adsorption and NOCNF Application Studies

The adsorption from capacity of NOCNF in suspension was estimated as follows. The ICP-MS results from the static Tl(I) adsorption study were used to calculate the *Q_e_* value and *C_e_/Q_e_* ratio (i.e., the original Tl(I) concentration divided by the adsorption capacity adsorbed by NOCNF at equilibrium). The value of *Q_e_* was calculated by multiplying the adsorption efficiency of NOCNF by the ideal adsorption capacity of NOCNF, based on the available carboxylate content (i.e., 0.69 mmol/g). The results from the Tl(I) adsorption experiments using the NOCNF adsorbent are summarized in Table S1 in Supporting Information. In this table, it was seen that the adsorption efficiency of NOCNF decreased from 99% to 92% when the Tl(I) concentration was increased from 10 ppm to 500 ppm.

**Figure 9:**
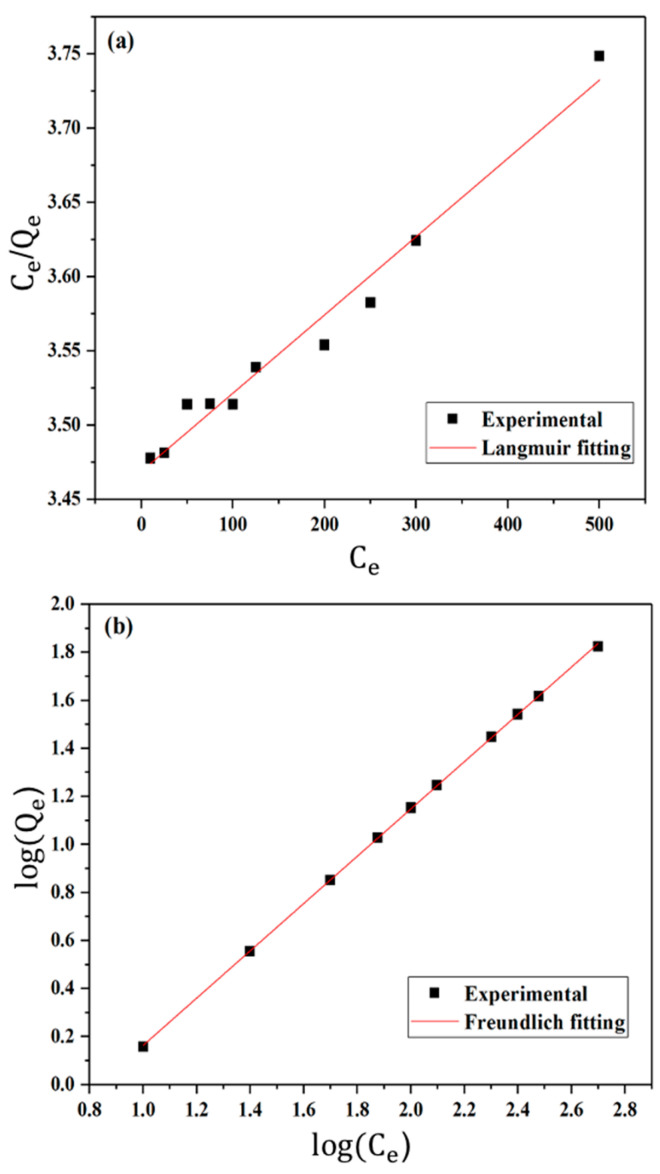
(**a**) Langmuir and (**b**) Freundlich isotherm fitting of the Tl(I) adsorption isotherm data by NOCNF (0.32 wt%).

The static Tl(I) adsorption results were fitted with both Langmuir and Freundlich models using Equations (1) and (2), respectively. In Figure 9a, the plot of *C_e_/Q_e_* versus Ce was illustrated using the Langmuir model, which exhibited a coefficient of determination R^2^ (or R-Squared) value of 0.975. In Figure 9b, the plot of log *Q_e_* versus log *C_e_* was illustrated using the Freundlich model, which exhibited an excellent R^2^ (or R-Squared) value of 0.999. The Freundlich model gave a better fit to the adsorption isotherm data, suggesting that the Tl adsorption mechanism is favored by the multilayer adsorption process. The parameters obtained from the Langmuir and Freundlich model fits of the Tl(I) adsorption isotherm data are summarized in Table 2.

**Figure 10:**
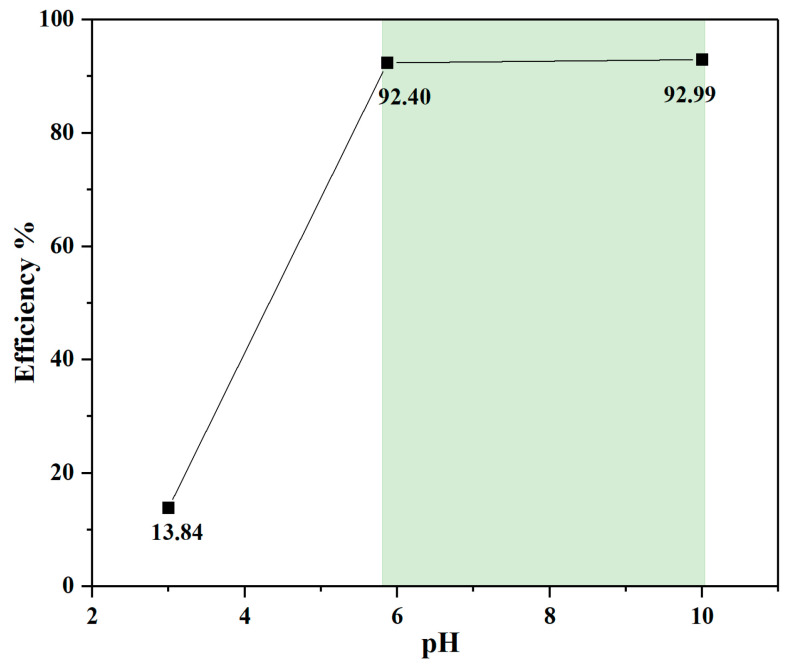
The pH study on the Tl(I) removal efficiency using NOCNF (0.32 wt%).

The pH effect on the Tl(I) removal efficiency using NOCNF was also investigated, where the results are illustrated in Figure 10. In this study, three different pH conditions: 3, 5.9 (drinking water) and 10, were considered. The ICP-MS results from the removal of Tl(I) at 500 ppm using the NOCNF adsorbent at three pH values are shown in Table S2 in Supporting Information and the corresponding plot of the removal efficiency versus the pH value is shown in Figure 10. It was found that the Tl(I) removal efficiency is very low (13.84%) when the pH level was 3. This is because the carboxylate group (COO-) is converted by to the carboxyl group (COOH) at the acidic condition, where no binding sites become available to adsorb Tl(I) ions. The high Tl(I) removal efficiency appeared at the neutral and basic conditions, (e.g., 92.4% at pH = 5.9 and 92.99% at pH = 10), indicating NOCNF can be used as a promising adsorbent for practical water purification conditions (as used for drinking water treatment).

**Figure 11:**
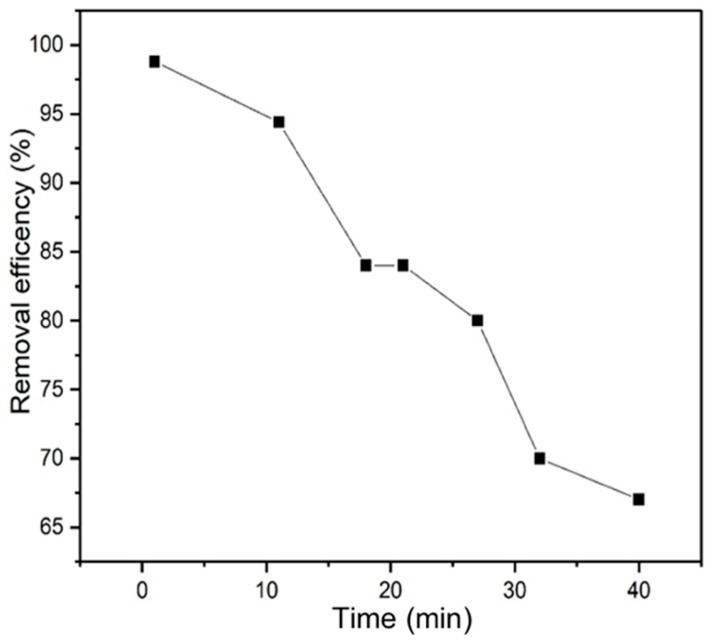
The Tl(I) removal efficiency using the freeze-dried NOCNF column test as a function of adsorption time.

To further explore the application of NOCNF for water purification, a column packed with freeze-dried NOCNF as described earlier (Figure 1) was evaluated. The Tl(I) removal efficiency results determined by ICP-MS from the column test using seven different columns (prepared at the identical conditions) are summarized in Table S3 in Supporting Information, where the plot of the removal efficiency versus time is shown in Figure 11. Notably, the flow rate (20 mL/min) of this test driving by gravity was kept almost the same. It was found that the initial Tl(I) removal efficiency of the column was 98.8% at the very beginning of the test, while it went down gradually to 67% after 40 min. This may be due to the decreases in the contact time and the carboxylate sites. It is clear that a slower flow rate and a higher concentration of available carboxylate groups on NOCNF will improve the overall Tl(I) removal efficiency.

### 3.4. Comparative Studies

Although the Langmuir model is not the best to describe the Tl(I) adsorption isotherm data by using NOCNF, the fitting of the Langmuir model can yield the *Q_m_* value of NOCNF (1898 mg/g). We have used this value to compare the Tl(I) removal capability by NOCNF with other adsorbents. This is because the studies of these adsorbents always used the Langmuir model to extract the maximum adsorption capacity. The comparison of the *Q_m_* value for Tl(I) removal by various adsorbents are listed in Table 3. This table included inorganic adsorbents (such as multiwalled carbon nanotubes [22], polyacrylamide-aluminosilicate composites [22] and nano-titanium dioxide [58]). It was seen that NOCNF extracted from sorghum stalks possessed the highest *Q_m_* value (almost twice of those for titanate nanotube- 709.2 mg/g [59] and nanosized manganese dioxide-672.7 mg/g [19]). Some natural products, such as sawdust samples were also included [25], however, their low removal capacities were due to the lower surface areas with limited adsorption sites (e.g., carboxylate groups).

## 4. Conclusions

A highly efficient adsorbent for Tl(I) removal was developed by extraction of carboxylated cellulose nanofiber directly from untreated sorghum stalks using the nitro-oxidation method, which is a simple, cost-effective, and zero-waste approach to produce nanocellulose. FTIR, SEM, TEM, AFM and WAXD characterizations of NOCNF and NOCNF-Tl floc samples confirmed that the Tl(I) removal is facilitated through the combinate effect of electrostatic interaction between Tl(I) and NOCNF and the mineralization of Tl_2_O_3_ in the NOCNF scaffold. As a result, NOCNF exhibited high removal efficiency (over 90% efficiency even in 500 ppm Tl(I) water) and high maximum adsorption capacity (*Q_m_* = 1898 mg/g) when compared to other adsorbents. The Tl(I) adsorption isotherm was fitted by both Langmuir and Freundlich models, while the latter gave a better fitting result. The preference of the Freundlich model (based on the hypothesis of multilayer physical adsorption) is consistent with the finding of Tl_2_O_3_ mineralization. The column adsorption test using freeze-dried NOCNF samples was carried out to investigate the dynamic adsorption for Tl(I) removal. It was seen that the performance of the freeze-dried NOCNF column was sufficiently good, making large scale Tl(I) removal using similar NOCNF adsorbent in a practical water treatment setting feasible.

## Figures and Tables

**Table 1:** Structure and surface properties of NOCNF extracted from sorghum stalks using the nitro-oxidation process.

	Carboxylate Content (mmol/g)	Total Lignin (%)	Residual Hemicellulose KL/AS (%)	Zeta Potential (mV)	Average Length (nm)	Average Width (nm)	Average Thickness (nm)
NOCNF	0.69	11.56	35	−110 ± 5	1390 ± 380	8.5 ± 2.0	1.6 ± 0.8

**Table 2:** Summary of parameters using Langmuir and Freundlich models to fit the Tl(I) adsorption isotherm data.

Adsorbent	Langmuir Model			Freundlich Model		
NOCNF	*Q_m_* (mg/g)	*K*	R^2^	*K_f_*	*n*	R^2^
	1898	1.519 × 10^−4^	0.975	0.151	1.016	0.999

**Table 3:** The comparison of the *Q_m_* value for Tl(I) removal by various adsorbents.

Adsorbents	*Q_m_* (mg/g)	Adsorption Range	Reference
Titanate nanotube	709	0–50 ppm	[59]
Nanosized manganese dioxide	672.7	0–10 ppm	[19]
Multiwalled carbon nanotubes	0.4		[23]
Polyacryamide–aluminosilicate composites	377.4	2–2000 ppm	[22]
Nano-titanium dioxide	51.2	0–20 ppm	[58]
Saw dust (untreated)	2.71	0–1021 ppm	[25]
Saw dust (treated)	13.2	0–1021 ppm	[25]
NOCNF-Sorghum stalks	1898	10–500 ppm	This study
Titanate nanotube	709.2	0–50 ppm	[59]

## Data Availability

Not applicable.

## Supplementary Materials

The following supporting information can be downloaded at: https://www.mdpi.com/article/10.3390/nano12234156/s1, Figure S1: Zeta potential and corresponding conductivity fluctuation of NOCNF; Figure S2: N_2_ adsorption results of NOCNF; Table S1: Tl(I) adsorption experiments data; Table S2: pH effect on the removak of Tl(I) by NOCNF; Table S3: Tl(I) removal data by freeze-dried NOCNF packed column. Reference [60] is cited in supplementary materials.
