# Supplementary material for: Effective Thallium(I) Removal by Nanocellulose Bioadsorbent Prepared by Nitro-Oxidation of Sorghum Stalks
Source: Nanomaterials (Basel). 2022 Nov 24;12(23):4156. doi: 10.3390/nano12234156 (PMC9740565; doi:10.3390/nano12234156)
Supplement: Supplementary file 1 [file nanomaterials-12-04156-s001.zip › nanomaterials-2059567-supplementary.pdf]

Supplementary materials

Effective Thallium(I) Removal by Nanocellulose Bioadsorbent Prepared by Nitro-Oxidation of Sorghum Stalks

Hui Chen [1], Priyanka R. Sharma [1], Sunil K. Sharma [1], Abdulrahman G. Alhamzani [2] and Benjamin S. Hsiao [1,*]

[1] Department of Chemistry, Stony Brook University, Stony Brook, NY 11794-3400, USA; huic1@umbc.edu (H.C.); priyanka.r.sharma@stonybrook.edu (P.R.S.); sunil.k.sharma@stonybrook.edu (S.K.S.)
[2] Department of Chemistry, Imam Mohammad Ibn Saud Islamic University (IMSIU), Riyadh 11632, Saudi Arabia; agalhamzani@imamu.edu.sa
* Correspondence: benjamin.hsiao@stonybrook.edu; Tel.: +16316327793

Fourier Transform Infra-Red Spectrometry (FTIR)

A PerkinElmer Spectrum One instrument was used to record the FTIR spectra between 450 and 4000 cm^{-1} in the transmission mode. A total of 6 scans were taken per sample with a resolution of 4 cm^{-1}. The solid samples were measured in the attenuated total reflectance (ATR) mode.

Thermogravimetric Analysis (TGA)

The thermal stability of NOCNF and NOCNF-floc was studied by a PerkinElmer STA-6000 instrument (Simultaneous Thermal Analyzer). Both TGA and differential thermogravimetry (DTG) curves were measured. The samples were run at a heating rate of 10 °C/min in the range of 0−800 °C under a continuous nitrogen flow.

Wide-Angle X-ray Diffraction (WAXD)

WAXD measurements were carried out using a Benchtop Rigaku MiniFlex 600 instrument. The samples were prepared by coating NOCNF suspensions on sample holders made of glass. The Cu Kα radiation was generated at 40 kV and 40 mA (λ = 0.154 nm) using a Ni filter. The data collection was carried out using a flat holder in the Bragg−Brentano geometry (diffraction angle = 5−50°; data was collected at a rate of 10°min^{-1}). The crystalinity of index (CI) of the NOCNF sample was estimated from the WAXD profile by using the

following equation:

$$CI = \frac{I(200) - I(am)}{I(200)} \qquad \text{(S1)}$$

where *I*(200) is the intensity of the dominant (200) diffraction peak, and *I*(am) is the intensity of the amorphous peak evaluated as the background peak located between the dominant (200) peak and the secondary (110) peak.[1]

Transmission Electron Microscopy (TEM)

TEM studies were carried out by a FEI Tecnai G2 Spirit BioTWIN instrument, operated at an accelerating voltage of 120 kV and equipped with a digital camera. The instrument also possessed the photographic film capability with goniometer and tilt stage accessories, as well as the electron diffraction capability. In typical sample preparation, a 10 μL aliquot sample of 1 mg of NOCNF in 10 mL distilled water was deposited on freshly glow discharged carbon coated Cu grids (300 mesh, Ted Pella Inc.).

Atomic Force Microscopy (AFM)

AFM measurements were performed using a Bruker Dimension ICON scanning probe microscope (Bruker Corporation, U.S.A.) equipped with a Bruker OTESPA tip (tip radius (max.) = 10 nm). A 10 μL of 0.005 wt % NOCNF suspension was deposited on the surface of a silica plate, where the air-dried sample was measured in the tapping mode.

Scanning Electron Microscopy (SEM)

The morphological study was recorded by a Zeiss LEO 1550 SFEG-SEM system. This SEM instrument consists of the standard E-T detector, as well as an In-Lens Secondary Electron Detector and a Rutherford Backscatter Electron Detector. The instrument was also equipped with an EDS (energy dispersive X-ray spectroscopy) unit having an EDAX detector, which provided elemental composition information and X-ray maps of the various phases of the materials examined.

Conductometric Titration Method

The carboxylate content (the ionic form) in NOCNF was determined by using the conductivity titration method. In this method, 0.3 g of dried nanofiber sample was dispersed in 55 mL of distilled water. The suspension was then set to a pH value in the range of 2.5−3 by adding 0.1 M HCl. Subsequently, 0.1 M NaOH was added to the suspension continuously until pH reached 11 (monitored by a pH meter), meanwhile, conductivity of the solution was recorded point by point during the titration process. The carboxylate content of NOCNF (the degree of oxidation) was calculated by using the following equation:

$$Content\ of\ carboxylate\ \left(\frac{mmol}{g}\right) = \frac{(V2-V1)*C(NaOH)}{mass\ of\ fibers} \qquad (S2)$$

Where V1 and V2 represent initial and finial NaOH volume showing in the bureau during the titration.

Inductively Coupled Plasma Mass Spectroscopy (ICP-MS)

Elemental concentration analyses were carried out on an Agilent 7500cx quadrupole ICP-MS instrument. Samples were diluted to generate signals that could match the mixed calibration standards, where unknown concentrations were calculated based on the standard calibration curves. The standard was run frequently between the unknown samples in order to monitor the drift in signal intensity of the instrument.

BET-Surface N_2 Adsorption Measurement

Surface area was measured using a Quantachrome NOVA touch LX^2 analyzer via a multi-point BET ((Brunauer, Emmet, and Teller) method. UHP-grade gases (N_2 and He) were used in the measurements without further purification. The N_2 adsorption measurements were carried out at 77 K in a liquid nitrogen bath. All samples were activated for 12 h at 110 °C under ultrahigh vacuum (10^{-8} mbar) built in the NOVA touch before proceding to gas adsorption measurements.

Rheology Measurement

Rheological measurements of different NOCNF-Tl flocs were conducted using an Anton Paar Physica MCR-301 rheometer with the concentric cylinder geometry (the operation gap was 1.12 mm). A steady shear were also carried out to estimate the viscosity of the

NOCNF-Tl floc in the frequency rage of 1 and 10 s^{-1}. A zero-shear viscosity was also estimated at the shear rate of 0.1 s^{-1} (measured for 16 min due to the machine sensitivity).

Lignin and Hemicellulose Content Measurement

The lignin and hemicellulose (total sugar) analysis of the samples (NOCNF from sorghum stalks) was performed by the Celignis Company in Ireland. The following analytical procedures were used in the analysis: (1) acid hydrolysis of samples (2) determination of acid soluble lignin (ASL) using UV-Vis spectroscopy (3) gravimetric determination of klason lignin (KL) (4) chromatographic analysis of hydrolyzate.

Table S1. The results from the Tl(I) adsorption experiments by the NOCNF adsorbent.

**Original Tl (ppm)**	**Remediation (2ml+2ml) (ppm) / C_e**	**After dilution (ppb)**	**ICP-MS (ppb)**	**Dilution**	**ICP-MS calculated (ppm)**	**Efficiency**	**Ideal adsorption capacity (mg/g)**	**Experimental capacity (mg/g) / Q_e**	C_e/Q_e
10	5	5	0.039	*1000	0.039	0.99	1.4	1.4	3.5
25	12.5	12.5	0.11	*1000	0.11	0.99	3.6	3.6	3.5
50	25	25	0.45	*1000	0.45	0.98	7.2	7.1	3.5
75	37.5	37.5	0.68	*1000	0.68	0.98	11	11	3.5
100	50	50	0.9	*1000	0.9	0.98	14	14	3.5
125	62.5	62.5	1.6	*1000	1.6	0.97	18	18	3.5
200	100	100	2.9	*1000	2.9	0.97	29	28	3.6
250	125	62.5	2.3	*2000	4.6	0.96	36	35	3.6
300	150	75	3.5	*2000	7	0.95	43	41	3.6
500	250	100	8.0	*2500	20	0.92	72	67	3.8

Table S2. The pH effect on the removal of Tl(I) at 500 ppm by the NOCNF adsorbent.

**pH**	**Original conc (ppm) (after mixture)**	**Final conc (ppm) (after conversion from ppb)**	**Efficiency (%)**
3	250	215.4	13.84
6	250	18.99	92.40
10	250	17.51	92.99

Table S3. The efficiency of Tl(I) removal using a freeze-dried NOCNF packed column.

**Time (min)**	**Column #**	**Initial (ppm)**	**Dilution*1000 (ppm)**	**Final (ppm)**	**Efficiency %**
1	1	100	0.1	0.0012	98.8
11	2	100	0.1	0.0056	94.4
18	3	100	0.1	0.016	84
21	4	100	0.1	0.016	84
27	5	100	0.1	0.02	80
32	6	100	0.1	0.03	70
40	7	100	0.1	0.033	67

Figure S1. Fluctuations of the zeta potential and corresponding conductivity curves of the NOCNF suspension (concentration = 0.32 wt%).

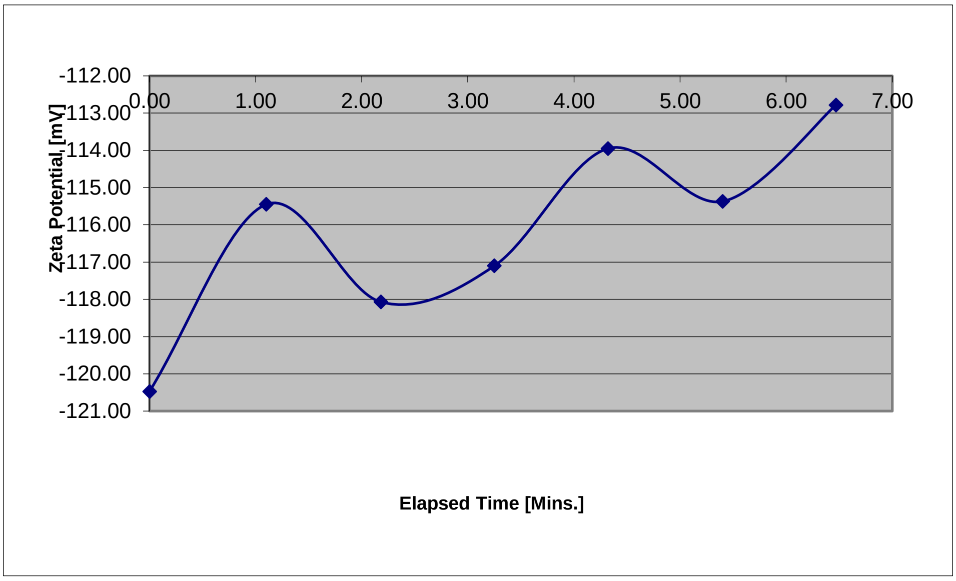

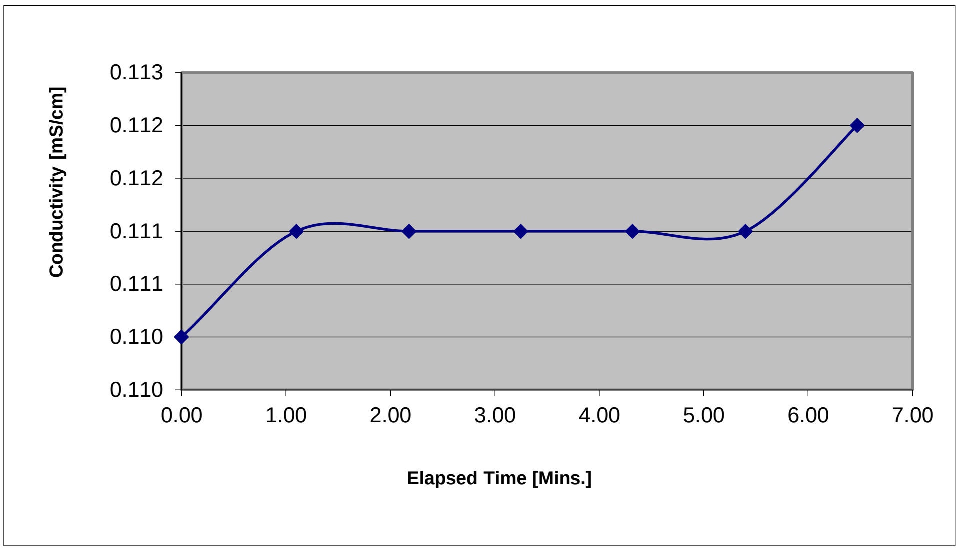

Figure S2. The N_2 adsorption results of the NOCNF adsorbent (extracted from sorghum stalks).

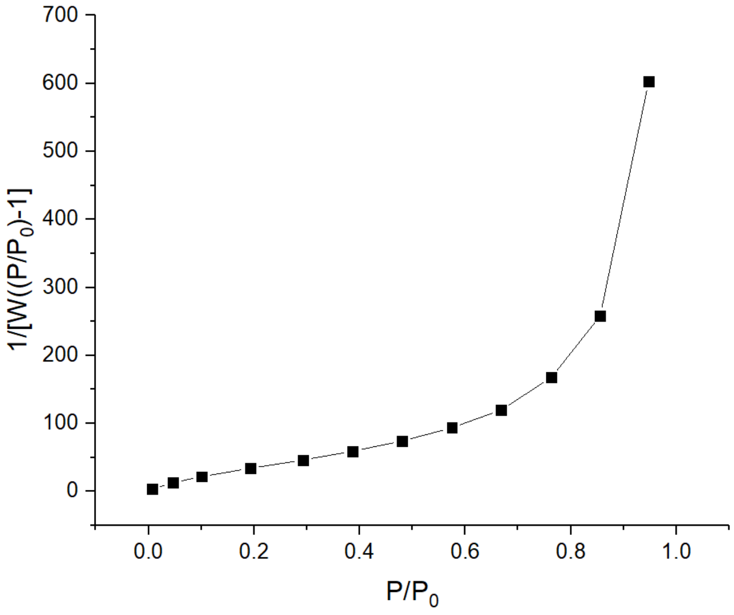

Reference

1. Segal, L.; Creely, J.J.; Martin, A.E., Jr.; Conrad, C.M. An Empirical Method for Estimating the Degree of Crystallinity of Native Cellulose Using the X-Ray Diffractometer. *Text. Res. J.* **1959**, *29*, 786–794.
